# Quantum-Dot-Based Iron Oxide Nanoparticles Activate the NLRP3 Inflammasome in Murine Bone Marrow-Derived Dendritic Cells

**DOI:** 10.3390/nano12183145

**Published:** 2022-09-10

**Authors:** Fernando Menegatti de Melo, Karine Kawasaki, Tarciso Almeida Sellani, Bruno Souza Bonifácio, Renato Arruda Mortara, Henrique Eisi Toma, Filipe Menegatti de Melo, Elaine Guadelupe Rodrigues

**Affiliations:** 1 Department of Chemistry, Institute of Chemistry, University of São Paulo (USP), Av. Lineu Prestes 748, Butantã, São Paulo 05508-000, SP, Brazil; 2 Metal-Chek do Brasil Indústria e Comércio, Research & Development Department, Rua das Indústrias, 135, Bragança Paulista 12926-674, SP, Brazil; 3 Department of Microbiology, Immunology and Parasitology, Federal University of São Paulo (UNIFESP), Rua Botucatu, 862, Vila Clementino, São Paulo 04023-062, SP, Brazil

**Keywords:** quantum-dot, iron oxide nanoparticles, NLRP inflammasome, dendritic cells

## Abstract

Inflammasomes are cytosolic complexes composed of a Nod-like receptor, NLR, the adaptor protein, ASC, and a proteolytic enzyme, caspase-1. Inflammasome activation leads to caspase-1 activation and promotes functional maturation of IL-1β and IL-18, two prototypical inflammatory cytokines. Besides, inflammasome activation leads to pyroptosis, an inflammatory type of cell death. Inflammasomes are vital for the host to cope with foreign pathogens or tissue damage. Herein, we show that quantum-dot-based iron oxide nanoparticles, MNP@QD, trigger NLRP3 inflammasome activation and subsequent release of proinflammatory interleukin IL-1β by murine bone marrow-derived dendritic cells (BMDCs). This activation is more pronounced if these cells endocytose the nanoparticles before receiving inflammatory stimulation. MNP@QD was characterized by using imaging techniques like transmission electron microscopy, fluorescence microscopy, and atomic force microscopy, as well as physical and spectroscopical techniques such as fluorescence spectroscopy and powder diffraction. These findings may open the possibility of using the composite MNP@QD as both an imaging and a therapeutic tool.

## 1. Introduction

Inflammasomes are cytoplasmic multi-protein complexes that recruit inflammatory caspases (via the CARD binding domain—caspase recruitment domain), such as caspase-1. This enzyme cleaves and promotes the maturation of pro-inflammatory cytokines such as interleukin-1β (IL-1β) and interleukin-18 (IL-18) by macrophages, dendritic cells, and some tumor cells, such as human melanomas [1,2]. The activation of inflammasomes begins with the activation of receptors from the NLR family (Nod-like receptor) or the HIN-200 family, such as AIM2 (absent in melanoma), in response to PAMPs (pathogen-associated molecular patterns), DAMPs (damage-associated molecular patterns) or other receptor-specific stimuli. Inflammasomes of four NLRs are the most studied: NLRP1, NLRP3, NLRP6, and NLRC4. NLRP3 and NLRP6 do not have the CARD domain in their structure, requiring the recruitment of the adapter protein called ASC (apoptosis-associated speck-like), which makes it possible to bind the CARD domain of caspases [2,3]. AIM2, like NLRP3 and 6, has no CARD domain, requiring ASC to bind caspases [4]. In this paper, we focus on the NLRP3 inflammasome. The mechanism of the NLRP3 inflammasome activation is not yet fully defined, as NLRP3 appears to be activated by different chemical species. However, a common event induced by these activators is a drop in intracellular K^+^ concentration.

The activation of the NLRP3 inflammasome can be divided into two phases: the priming phase and the assembly phase. In the priming phase, a first molecular signal is provided by a TLR ligand (TLR-2, 3, 4 or 7) or by TNF-α; the main consequence of this phase is the nuclear factor κappa B (NF-κB)-dependent transcriptional activation of pro-IL-1β and NLRP3. In the assembly phase, the second signal is provided by diverse chemical species like ATP (by binding to the P2X7 receptor), Nigericin, and urate crystals, among others [5]. Unlike NLRP3, AIM-2 and NLRC4 do not have their transcription increased after macrophage activation with LPS; moreover, translation of NLRP3 is essential for the activation of this inflammasome, as cycloheximide-treated macrophages do not show caspase-1 activation when stimulated with Nigericin (NLRP3 agonist), whereas flagellin (NLRC4 agonist) and poly dA:dT (AIM-2 agonist) normally promote caspase-1 cleavage [6,7].

NLRP3 inflammasome proved crucial for protection against several pathogenic microorganisms, such as *Salmonella typhimurium* [8] and *Leishmania* spp. [9]. On the other hand, chronic or dysregulated activation of NLRP3 inflammasome has been shown to play a role in the progression of inflammatory diseases like gout [10], multiple sclerosis [11], and cryopyrin-associated autoinflammatory syndrome (CAPS) [12]. Regarding tumors, conflicting experimental evidence points to detrimental or protective roles depending on the context. For example, some authors have shown that the NLRP3 inflammasome is constitutively activated in metastatic melanoma, and the resulting IL-1β may play a role in stimulating angiogenesis [13]. Others have also shown that NLRP3 contributes to the expansion of myeloid-derived suppressor cells (MDSC) in melanoma patients [14].

On the other hand, Ghiringhelli et al., 2009, showed that dying tumor cells release ATP that activates the NLRP3 inflammasome in dendritic cells (DCs). The consequential release of IL-1β is essential for the efficient priming of IFN-γ-secreting T CD8+ lymphocytes and, therefore, an effective adaptative immune response against breast cancer [15]. Accordingly, in the mouse melanoma model B16F10, it has been recently shown that IL-1β administration to the host may exert adjuvant effects on the expansion and cytotoxic capacities of T cells in patients that are irresponsive (or poorly responders) to adoptive cell therapies (ACTs) [16]. The effects of IL-1β have not only been addressed on tumor immunotherapy, however. Recently, interesting experimental evidence points out that pyroptosis can contribute to immune-mediated tumor rejection, even if this type of cell death occurs in a small fraction of the cell population [17]. Considering these findings, we evaluated the impact a designed multifunctional magneto-fluorescent nanoparticle could have as both a suitable inflammasome activator and as a biological staining tool.

In recent years, a variety of magnetic nanoparticles (MNP) have been broadly applied in several fields as probes to identify fractures in ferromagnetic surfaces [18,19] as NMR contrast agents [20], in drug delivery [21], cell imaging [22,23], and nanohydrometallurgy [24,25,26]. However, designing and producing multifunctional MNP with tailored magneto-fluorescent properties with a specific application purpose has been challenging. Functional MNP has been synthesized primarily by modifying functional groups at the surface of the magnetic nanoparticles or combining them with other functional materials, such as quantum dots (QD), which are settled as biological imaging agents [27,28,29]. It can be treated with non-steroidal anti-inflammatory drugs to target specific tissues or cell organelles [30,31]. Besides, one of the most attractive properties is that QD emission spectra can be tuned by varying particle size or surface composition. Furthermore, quantum dots that emit at several different wavelengths can be excited with a single wavelength, are more resistant to bleaching, and have excellent stability under harsh conditions [27].

Herein, fluorescent water-based cadmium telluride quantum dots (QD) and citrate-functionalized maghemite nanoparticles (MNP) were synthesized and assembled (MNP@QD) through electrostatic forces by cetyltrimethylammonium bromide (CTAB) side chain interaction as previously reported [18], triggering NLRP3 inflammasome activation and subsequent release of proinflammatory interleukin IL-1β by murine bone marrow-derived dendritic cells.

## 2. Materials and Methods

### 2.1. Chemicals and Other Materials

Ferrous chloride tetrahydrate (FeCl_2_·4H_2_O), ferric chloride hexahydrate (FeCl_3_·6H_2_O), aqueous ammonia (NH_3(aq)_), citric acid, 3-mercaptopropionic acid (3 MPA), tellurium at 200 mesh (Te), sodium (Na), naphthalene (C_10_H_8_), and cetyltrimethylammonium bromide (CTAB) were employed as supplied from Sigma/Aldrich, SP, Brazil (new bottles, kept closed in desiccators, analytical grade). Cadmium acetate (Cd(OAc)), sodium hydroxide (NaOH), tetrahydrofuran (THF), and acetone (C_3_H_6_O) were employed as supplied from Labsynth, SP, Brazil. All cell culture reagents (cell media, supplements, Trypsin-EDTA) were acquired from ThermoFisher Scientific, SP, Brazil. All plasticware for cell culture was acquired from Corning, SP, Brazil. Mouse recombinant GM-CSF was acquired from R&D Systems, SP, Brazil, while Ultrapure LPS (from *E. coli* 0111:B4) and Nigericin were acquired from Invivogen, SP, Brazil. 4′, 6-diamidino-2-phenylindole (DAPI), glycerol-p-phenylenediamine, and all remaining salts and solutions were acquired from Sigma-Aldrich, SP, Brazil. APC-conjugated anti-mouse CD11c and fluorescein-5-isothiocyanate (FITC)-conjugated anti-mouse MHC Class II I-Ab antibodies were acquired from ThermoFisher Scientific, SP, Brazil. Cytokine ELISA were from BD Biosciences, SP, Brazil.

### 2.2. Synthesis of Sodium Telluride (Na_2_Te_x_)

At first, 6.1 g (48 mmol) tellurium at 200 Mesh, 2.2 g sodium (96 mmol), and 0.1 g (0.8 mmol) naphthalene were weighed, then 50 mL Schlenk flask was added. Then, 20 mL THF was mixed and stirred for 24 h at 60 °C. After that, the reaction medium was filtered, and the crude product was washed with 20 mL THF and dried under a vacuum.

### 2.3. Synthesis of Citrate-Functionalized Maghemite Nanoparticles (MNP)

The citrate-functionalized maghemite nanoparticles were synthesized as reported before [18]. The process was based on the modified co-precipitation method, starting from 15 mL of NH_3_ solution in a 125 mL three-neck round-bottom flask equipped with mechanical stirring. Then, 35 mL of deaerated water was added, and N_2_ flow was passed at 0.5 L min^−1^ (Flask 01). In another round-bottom glass, ferrous chloride tetrahydrate (0.63 mmol) and ferric chloride hexahydrate (1.26 mmol) were dissolved in 25 mL of deaerated water (Flask 02). Adding flask 02 content into flask 01 by applying a pressure difference, 10 mL of citric acid solution (0.5 mol L^−1^) was added, and the mechanical stirring was stopped after 1 h. The black magnetic nanoparticles were confined by using a Nd_2_Fe_14_B magnet, and it was washed twice (stock suspension). After lyophilization, the solid was kept dry in the desiccator, at room temperature, for characterization only. The previous stock suspension (50 mL) was put in a three-neck round-bottom flask (125 mL) equipped with a condenser and mechanical stirring under air bubbling. After starting the reflux, 5 mL of citric acid solution (0.5 mol L^−1^) was added and maintained for 150 min. The brown magnetic nanoparticles were confined to the bottom, and after removing the suspension, the solid was washed twice.

### 2.4. Synthesis of Fluorescent Water-Based Cadmium Telluride Quantum Dot (QD)

In a three-neck round-bottom flask, 100 μL of 3 MPA was added to a 5 mmol L^−1^ Cd(OAc). Using 1.0 mol L^−1^ NaOH, the pH was adjusted to 9.0. Then, this solution was deoxygenated for 30 min and heated up to 98 °C. After that, a 2.5 mmol L^−1^ Na_2_T_x_ aqueous solution was prepared and added abruptly to the Cd(II)/ligand solution. The reaction proceeded for 140 min.

### 2.5. Assembling MNP with QD (MNP@QD)

**Figure 1:**
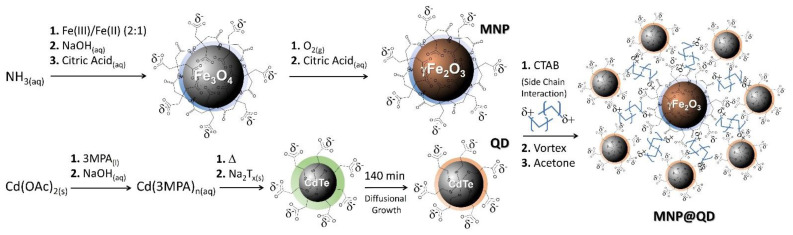
Illustrative representation of citrate-functionalized maghemite nanoparticles (MNP) and fluorescent water-based cadmium telluride quantum dots (QD) synthesis and its assembling with cetyltrimethylammonium bromide (CTAB) by electrostatic and side chain interactions.

A desired amount of MNP was dispersed in water (2 mL, 0.4 g L^−1^). It was added to CTAB solution (1 mL, 10 g L^−1^) and stirred in a vortex equipment for 10 s. At the end, 2 mL of the QDs suspension was added and the mixture was kept under gently mechanical stirring for 30 min, and the particles were precipitated with acetone and stocked. Several aqueous solutions were prepared for the inflammasome activation studies. Figure 1 summarizes the whole process of the MNP@QD synthesis.

### 2.6. Characterization of MNP, QD, and MNP@QD

The fine particle morphology was analyzed by high resolution transmission electron microscopy (HRTEM) on JEOL, model JEM 2100, operating with a LaB6 filament, with an acceleration voltage of 200 kV, using a drop casting technique and by scanning probe microscopy (SPM) using a PicoSPM I equipment, with a PicoScan2100 controller, and a MACMode setup from Molecular Imaging, using a drop casting technique too under mica. The fluorescence image of QD was obtained with the help of an inverted fluorescence microscope (Axiovert 200, Carl Zeiss, SP, Brazil) equipped with a Plan-Apo 63x/1.4 NA oil-immersion objective. The number 10 filter set (Carl Zeiss, SP, Brazil) was used, providing an excitation bandpass from 450 to 490 nm and an emission bandpass from 515 to 650 nm. Images were recorded on an Axiocam HSm digital camera (Carl Zeiss, SP, Brazil) and processed using the ZEN software (Carl Zeiss, SP, Brazil). Magnetization curves were obtained using a magnetometer manufactured by EG&G Princeton Applied Research, Gaithersburg, Maryland, US, model 4500, using magnetic fields of up to 20 kOe (Walker Scientific, Worcester, Massachusetts, US, HR8 model and the Gauss meter provided by Lake Shore, Westerville, Ohio, US, model450). The diffraction patterns were obtained on a Bruker D8 Discover facility. The measurement was obtained with a primary twin (0.6 mm; primary axial Soller) and a secondary twin (maximum aperture 5 mm; secondary axial Soller and nickel filter in 1D mode), with 0.05 as an increment and 1.5 as a step from 20 to 70°. Electronic absorption spectra were recorded on a diode-array Hewlett-Packard 8453A spectrophotometer, and photoluminescent emission was recorded on Photon Technology equipment, using an InGaAs detector and FelixGX 1317 software. The statistical size distribution was obtained by counting 300 nanoparticles using ImageJ 1.53k

### 2.7. Real-Time Kinetics of QD Formation

As previously reported, the real-time kinetics of QD formation was monitored using MasterView^®^ software [18]. Typically, commercial webcams are used to collect transmitted and reflected light from the reaction system, since incandescent lamps with emission maxima between 600 and 800 nm are used as the illumination source. From these signals, the software stores the averaged data from the red, green, and blue channels as a function of time in a predetermined spatial domain. As the nanoparticles are formed, light is absorbed and scattered at different wavelengths, depending on the average particle size and composition. Therefore, the RGB number increases or decreases depending on the content of scattering and absorbing materials in the three-necked flask, so we can obtain specific QDs without disturbance.

### 2.8. Murine Melanoma Cell Line B16F10-Nex2

B16F10-Nex2 lineage, which is syngeneic to C57BL/6 mice, was obtained from Banco de Células (BCRJ), RJ, Brazil. Tumor cells were cultured in RPMI-1640 medium supplemented with 10% heat-inactivated fetal bovine serum (FBS), 24 mmol L^−1^ NaHCO_3_, 10 mmol L^−1^ HEPES (4-(2-hydroxyethyl)-1-piperazineethanesulfonic acid), and 40 mg L^−1^ gentamicin. Cells were maintained in incubators at 37 °C under an atmosphere containing 5% CO_2_. Cells were detached from culture flasks with sterile phosphate saline buffer (PBS: 140 mmol L^−1^ NaCl, 2.7 mmol L^−1^ KCl, 10 mmol L^−1^ Na_2_HPO_4_, 1.8 mmol L^−1^ KH_2_PO_4_, pH 7.4) containing 2mM Na_2_EDTA (Ethylenediaminetetraacetic Acid, Disodium Salt, Dihydrate).

### 2.9. Bone Marrow-Derived Dendritic Cells (BMDCs) Differentiation

Bone marrow was obtained from male C57BL/6 mice, 8 weeks old. Mice were acquired from Centro de Desenvolvimento de Modelos Experimentais (CEDEME, UNIFESP) and maintained in a vivarium in specific pathogen-free (SPF) conditions, with water and food provided *ad libitum* and under a 12-h light/dark cycle. Animals were euthanized with a lethal intraperitoneal injection of ketamine (240 mg/kg) and xylazine (30 mg/kg) before the aseptic surgical removal of posterior members. All procedures were approved by the Committee on the Ethics of Animal Experimentation (CEUA/UNIFESP 9134290618). Bones were cleaned from muscles and other tissues, and the epiphyses were cut to allow the washout of bone marrow cells with sterile PBS. Red blood cells were lysed with ACK buffer (150 mmol L^−1^ NH_4_Cl, 10 mmol L^−1^ KHCO_3_, 0.1 mmol L^−1^ Na_2_EDTA). After hemolysis, cells were washed with sterile PBS and cultured for nine days in high glucose Dulbecco’s Modified Eagle Medium (DEMEM) supplemented with 10% FBS, 50 mmol L^−1^ 2-mercaptoethanol, 1 mmol L^−1^ sodium pyruvate, 100 U/mL penicillin, 10 mg/mL streptomycin, vitamins, non-essential amino acids, and 20 ng/mL mouse recombinant granulocyte-macrophage colony-stimulating factor (GM-CSF). Fresh media was added on days four and seven. After the nine days of differentiation, adherent and non-adherent cells were collected in cold PBS with the help of a cell scraper. The degree of differentiation was monitored by Flow Cytometry in a FACS Canto II (BD Biosciences) platform, and only cultures that showed more than 90% of CD11c^+^ MHC Class II I-Ab^+^ cells were used in experiments.

### 2.10. Toxicity of MNP@QD in B16F10 and BMDC

B16F10-Nex2 were seeded in 96-well plates at the density of 4.0 × 10^3^ cells/well in RPMI-1640 supplemented with 10% FBS. BMDCs were seeded at the density of 1.0 × 10^5^ cells/well. After a 24 h resting period to allow the cells to adhere, the media was replaced with fresh media containing different concentrations of MNP, QD, and MNP@QD. Cells were incubated for 24 h and 48 h after nanoparticle addition. After incubation, cells were detached with Trypsin-EDTA 0.25% solution, and viable cells were quantified in Neubauer chambers with the Trypan blue vital stain. Cell viability was evaluated in different temperature (4 °C or 37 °C) and pH (6.0 or 7.0) conditions. A colony-forming assay (without agar) was also performed with the tumor cells. B16F10-Nex2 were seeded in 6-well plates at the density of 1.0 × 10^2^ cells/well in RPMI-1640 supplemented with 10% FBS. Twenty-four hours later, cells were treated with the nanoparticles for more 24 h; after that, the media was replaced with fresh media, and cells were cultured for ten days to allow the colonies to develop. Colonies were fixed with 4% formaldehyde plus 0.5% (*w*/*v*) crystal violet in H_2_O.

### 2.11. Cytokine Production by BMDC

BMDCs were seeded in 96-well plates at 1.0 × 10^5^ cells/well density. After 24 h of resting, cells were treated with MNP@QD as described in Figures. Positive controls consisted of cells treated with 200 ng/mL LPS for 24 h for the induction of IL-6, TNF-α, and IL-12p70. For NLRP3 inflammasome activation and IL-1β secretion, cells were primed with 200 ng/mL LPS for 3 h and then activated with 5 μmol L^−1^ Nigericin for 30 min. MNP@QD was added at different time points of the inflammasome activation protocol to evaluate its regulatory capacity on the production/secretion of IL-1β. All cytokines were quantified in culture supernatants by ELISA according to manufacturer’s instructions.

### 2.12. Fluorescent Detection of MNP@QD in the Cytoplasm of BMDCs

Coverslips were sterilized and treated with 0.01% poly-lysine for 30 min. The slides were then washed with sterile PBS, put into wells from a 24-well plate, and 4.0 × 10^5^ BMDCs were seeded over the glass surface. Cells were cultured in RPMI-1640 with 10% FBS and treated with aggregated MNP@QD. To induce aggregation, the stock solution of 1.0 × 10^−6^ mol L^−1^ MNP@QD was treated with 1.0 mol L^−1^ NaCl (1:1, *v/v*). This solution was diluted the same way we performed with the native nanoparticles (that is, the dilutions that were necessary to achieve 10^−9^ M and 10^−8^ mol L^−1^ of the native MNP@QD) in cell culture media, and BMDCs were treated with the aggregated nanoparticles for 24 h. Recovered coverslips were washed with PBS, fixed with 4% paraformaldehyde, permeabilized with 0.1% saponin, and stained with 4′, 6-diamidino-2-phenylindole (DAPI) for 20 min. The coverslips were mounted on slides with 1 mM glycerol-p-phenylenediamine buffer before evaluation using epifluorescence (BX51; Olympus, Tokio, Japan) or confocal (TCS SP5 II Tandem Scanner; Leica, Wetzlar, Germany) microscopes. Images were acquired with 1000× magnification and were further processed with Imaris 7.0.0 (Bitplane, Belfast, UK) e ImageJ (NIH, Bethesda, MD, USA) software.

### 2.13. Statistical Analysis of Cellular Experiments

Comparisons among three or more groups were performed with either One-Way or 2way Analysis of Variance (ANOVA) with Sidak’s or Dunnet’s post hoc tests, respectively. Differences were considered statistically significant if *p* < 0.05. Data were plotted and analyzed with GraphPad Prism (Graph Pad Software, San Diego, CA, USA). Legend: * *p* < 0.05; ** *p* < 0.01; *** *p* < 0.001; **** *p* < 0.0001.

## 3. Results

### 3.1. Synthesis and Characterization of MNP@QD

**Figure 2:**
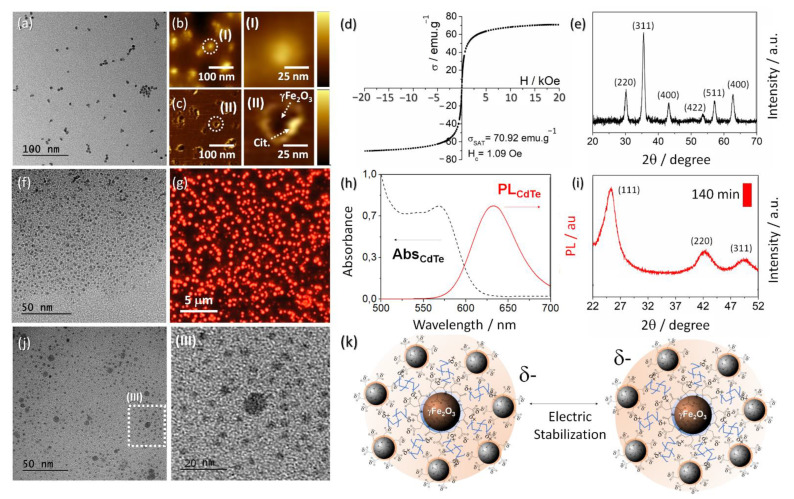
(**a**) TEM micrograph of MNP. (**b**,**c**) AFM topography and phase contrast images, respectively, of induced-agglomerated MNP showing the interaction among iron oxide nanoparticles and the existence of exposed citrate at the nanoparticles surface. (I, II) Inset: digital zoom-in from b and c, respectively. (**d**) Magnetization curve of MNP. (**e**) Powder diffraction of MNP with diffraction planes assigned. (**f**) TEM micrograph of QD. (**g**) Fluorescence image of aggregated QD obtained by an inverted fluorescence microscope. (**h**) Absorption and emission spectra of QD under 470 nm excitation. (**i**) Power diffraction of QD after 140 min reaction. (**j**) TEM micrograph of MNP@QD. (III) Inset: digital zoom-in from j. (**k**) Pictorial representation of the electric stabilization of the composite.

The demand for new materials has always driven humanity to progress in the most diverse areas of knowledge, providing what is necessary to develop new products that improve quality of life. Despite combining two or more functional constituents into one material, creating the so-called multifunctional materials that can be applied to a wide range of advanced technological uses, their development remains quite challenging. Herein, citrate-functionalized maghemite nanoparticles were exploited as the anchoring surface to the fluorescent water-based cadmium telluride quantum dot by using cetyltrimethylammonium bromide as previously reported [18]. Figure 2 shows the main characterization of the MNP@QD. Real-time kinetics of QD formation and statistical size distribution of MNP, QD, and MNP@QD are shown in Supplementary Material, Figures S1 and S2.

The large-area transmission electron microscopy, TEM images, in Figure 2a,f,j show that the resulting quasi-spherical nanoparticles, MNP, QD, and MNP@QD, have an average diameter of 12.5 ± 3.1 nm, 8.5 ± 2.2 nm, and 43.5 ± 4.8 nm, respectively. As previously reported [18,19], the oxidation process can be assisted by Raman spectroscopy and SAED (specific area electron diffraction). The powder diffraction of the MNP has indicated the spinel structure and the successful oxidation by the (511) shifts [19]. In addition, VSM measurement, Figure 2d, showed the maintenance of superparamagnetic behavior after the oxidation process, since the coercivity field is lesser than 12.5 Oe and the high loading magnetic content after oxidation. As previously reported [18], the saturation magnetization of the composite is usually preserved after the QD attachment at the iron oxide nanoparticles surface. Using two standard samples, Fluorescein and Rhodamine 6G, we calculated an average quantum yield to QD as 81% and to MNP@QD as 75%. In that case, PL emission band is not so broad, varying mainly from 630 nm to 680 nm, as shown in Figure 2h. Besides, this QD has a cubic structure containing three major peaks, 111, 220 and 311, confirming its good crystallinity and purity, Figure 2i. As shown in Figure 2j, the MNP@QD is homogeneous and must be stored in acetone solution to avoid destabilization since electrostatic interactions assure its integrity. There are a few QD outside the composite sphere, and it is probably related to the drying process of the TEM grid for imaging.

### 3.2. The MNP@QD Is Non-Toxic to Melanoma and Dendritic Cells under Physiological pH

**Figure 3:**
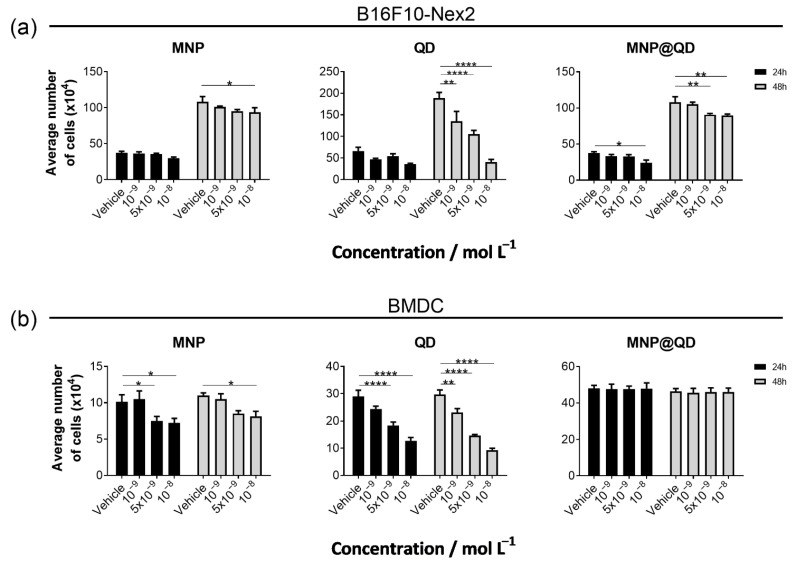
Toxicity evaluation of MNP, QD, and MNP@QD in melanoma cells (**a**) and BMDCs (**b**) after 24 h and 48 h with each nanoparticle. Cell viability was assessed with the Trypan Blue method. Each bar represents the average of five replicates. Error bars represent the standard error of the mean (SEM). Graphs are representative of three independent experiments. * *p* < 0.05; ** *p* < 0.01; **** *p* < 0.0001. Statistical significance was obtained with 2way ANOVA with Dunnet’s multiple comparisons test.

B16F10 melanoma cells and bone marrow-derived dendritic cells, BMDCs, were treated with increasing concentrations starting from 10^−9^ mol L^−1^ of the MNP@QD or its nanoparticle subunits (MNP and QD) for 24 h and 48 h. After these periods of incubation, we evaluated cell viability. We chose to study the effect of the nanoparticles on dendritic cells because they are the primary antigen-presenting cells (APCs) to T-lymphocytes. Several immunotherapeutic strategies against tumors target these cells in the tumor microenvironment or in draining lymph nodes [31,32]. The MNP@QD is generally non-toxic to both cell types, with discrete toxicity observed when the cells were treated with 5 × 10^−9^ and 10^−8^ mol L^−1^ for 48 h, Figure 3a. Regarding the B16F10 cells, MNP was non-toxic, while the QD was highly toxic even at 10^−9^ mol L^−1^, Figure 3a. The toxicity results correlated well with what we observed in colony-forming assays, as shown in Supplementary Materials, Figure S3. While the MNP@QD moderately inhibited the colony forming at the 5 × 10^−8^ and 10^−8^ mol L^−1^ doses, QD highly inhibited the colony forming, while MNP showed no effect. Regarding the BMDCs, the MNP@QD was well tolerated in both time points in all doses tested, Figure 3b. MNP was slightly toxic at higher concentrations, and QD was highly toxic in all concentrations. These results show that incorporating QD in MNP@QDs significantly reduces the toxicity of these nanoparticles, mainly because the QD are trapped at the nanoparticle’s surface and are not available to the cell environment, making them feasible to be used as multifunctional nanoparticles in tumor immunotherapy. The dose of 10^−9^ mol L^−1^ is the most tolerated by both cell types.

**Figure 4:**
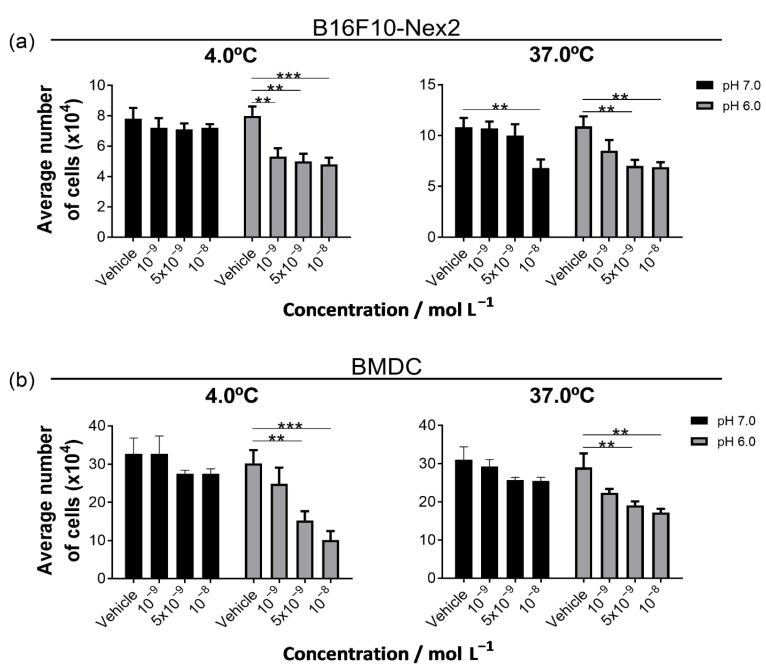
Toxicity evaluation of MNP@QD in melanoma cells (**a**) and in BMDCs (**b**) cultured either at pH 7.0 or pH 6.0, at 4 °C and 37 °C. Cell viability was assessed with the Trypan Blue method. Each bar represents the average of five replicates. Error bars represent SEM. Graphs are representative of three independent experiments. ** *p* < 0.01; *** *p* < 0.001 Statistical significance was obtained with 2way ANOVA with Dunnet’s multiple comparisons test.

Due to the metabolic shift accompanying tumor development, the tumor microenvironment generally acquires an acidic condition. This acidification tends to interfere with immune cell activation and chemotherapeutic drugs’ pharmacokinetics [26]. We evaluated the toxicity of the MNP@QD on both B16F10 cells and BMDCs at pH = 6.0 and pH = 7.0. Additionally, we experimented with two incubation temperatures, 4 °C and 37 °C, to evaluate the dependency of active metabolism on the toxicity effect. For B16F10 cells, the toxicity exerted by the MNP@QD at the 10^−8^ mol L^−1^ concentration is abolished at 4 °C. Regarding the pH effect, the toxicity increases at pH = 6.0 at both temperatures. The 10^−9^ mol L^−1^ dose becomes toxic at acidic pH, a more prominent phenomenon at 4 °C, Figure 4a. For the BMDCs, there is no toxicity either at 4 °C or 37 °C if cells are incubated at pH = 7.0. On the other hand, there is statistically significant toxicity for the higher concentrations (5 × 10^−9^ and 10^−8^ mol L^−1^) at pH = 6.0. We also observe a tendency of toxicity if the cells are treated with 10^−9^ mol L^−1^, Figure 4b. Taken together, we conclude that acidic conditions increase the toxicity of the MNP@QD. This increase might be explained by the fact that acidic conditions promote the nanoparticles’ protonation, which tends to neutralize its negative charges and contributes to the passive entrance of the particles into the cells. The precise mechanism of induction of cell death in these conditions is being investigated.

### 3.3. MNP@QD Does Not Induce IL-6, TNF-α, and IL-12p70 Production Alone but Slightly Enhances LPS-Induced Cytokine Secretion

**Figure 5:**
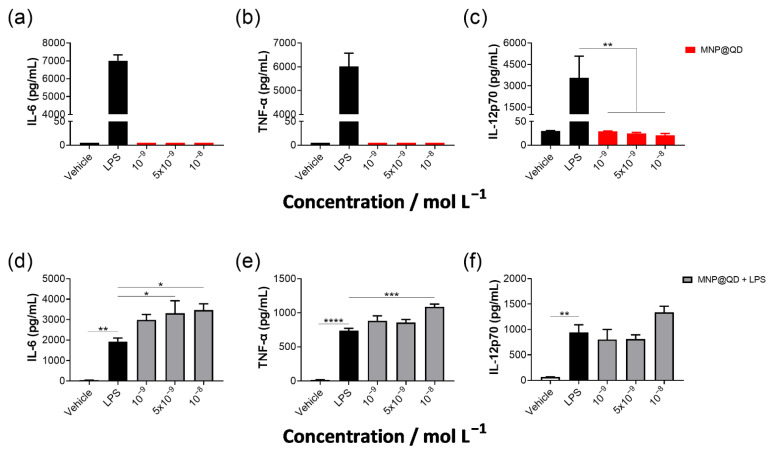
Cytokine production by BMDCs treated with the MNP@QD. IL-6, TNF-α, and IL-12p70 secretion after 24 h-treatment with the MNP@QD alone (**a**–**c**) or after a 24 h-treatment with the MNP@QD plus 24 h LPS treatment (**d**–**f**). Cytokine production was evaluated in culture supernatants by ELISA. Each bar represents the average of four replicates. Error bars represent SEM. Graphs are representative of three independent experiments. * *p* < 0.05; ** *p* < 0.01; *** *p* < 0.001; **** *p* < 0.0001. Statistical significance was obtained with One-way ANOVA with Sidak’s multiple comparisons test.

We also investigated the activating potential of the MNP@QD on BMDCs by evaluating cytokine production. None of the tested concentrations induced the secretion of IL-6, TNF-α, and IL-12p70 when cells were treated with the MNP@QD alone, Figure 5a–c. However, the MNP@QD enhanced LPS-induced secretion of IL-6, although the difference was only statistically significant at the concentrations of 5 × 10^−9^ and 10^−8^ mol L^−1^. Regarding TNF-α, statistical significance was only observed at the higher concentration, while the production of IL-12p70 did not seem to be modulated by the presence of the nanoparticles, Figure 5d–f.

### 3.4. MNP@QD Enhances IL-1b Secretion

Inflammasomes are macromolecular platforms expressed by several types of cells, including cells derived from the myeloid lineage, like macrophages and dendritic cells. These molecular platforms are critical for the protection of the host against some intracellular microorganisms. The mechanisms involved in the protection include the secretion of IL-1β and IL-18 and the activation of pathways that lead to an inflammatory type of cell death called pyroptosis. Cytokines and pyroptosis mediators are activated by proteolytic cleavage by caspase-1 [33]. Since it has been long understood that nanoparticles can activate inflammasomes once they reach the phagocytic pathway [34], we decided to evaluate if the MNP@QD could activate the NLRP3 inflammasome by measuring the secretion of IL-1β.

**Figure 6:**
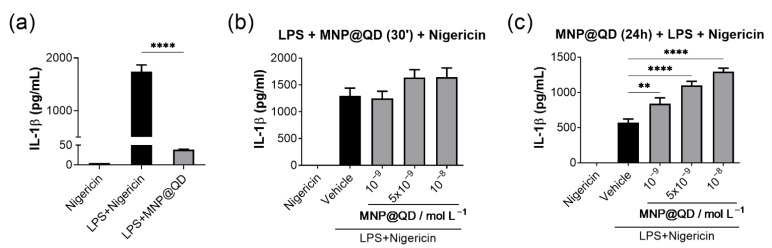
IL-1β production by BMDCs. (**a**) Cells were primed with LPS for 3 h and treated with Nigericin or 10^−8^ mol L^−1^ MNP@QD for 30 min. (**b**) BMDCs were treated with MNP@QD for 30 min after the 3 h-priming with LPS. Then, cells were treated with Nigericin for an additional 30 min. (**c**) BMDCs were incubated with MNP@QD for 24 h, then primed with LPS (3 h) and treated with Nigericin (30 min). IL-1β production was evaluated in culture supernatants by ELISA. Each bar represents the average of four replicates. Error bars represent SEM. Graphs are representative of two independent experiments. ** *p* < 0.01; **** *p* < 0.0001. Statistical significance was obtained with One-way ANOVA with Sidak’s multiple comparisons test.

Cells activated with LPS + MNP@QD produced marginal amounts of IL-1β compared to those activated with LPS + Nigericin, Figure 6a. Then, we tried to determine if the incubation with the MNP@QD could modulate the secretion of IL-1β induced by LPS + Nigericin. BMDCs were primed with LPS for 3 h, treated with the same doses of MNP@QD previously tested (10^−9^, 5 × 10^−9,^ and 10^−8^ mol L^−1^) for 30 min, and then treated with Nigericin for 30 min. None of the MNP@QD concentrations produced a statistically significant effect on IL-1β secretion, Figure 6b. Finally, to increase the endocytosis of the nanoparticles by the DCs, cells were pre-incubated with the nanoparticles for 24 h before the LPS priming (3 h) and Nigericin activation (30 min). In this scenario, the nanoparticles enhanced the secretion of IL-1β compared to cells that received only LPS + Nigericin, Figure 6c. These results show that the MNP@QD can augment the secretion of IL-1β under certain conditions, mainly if the myeloid cells have endocytosed the particles before receiving inflammatory stimuli.

### 3.5. Fluorescent Detection of MNP@QD in BMDCs

We first performed fluorescence microscopy to verify if the MNP@QD is indeed internalized by the dendritic cells. The QD that is complexed in the MNP@QD emits fluorescence if excited by the range of wavelengths between ultraviolet (365 nm) and red light (>635 nm). Since conventional fluorescence microscopy has a resolution of 200 nm and the MNP@QDs have an average diameter of 45 nm, we decided to treat the MNP@QD stock solution (concentration = 10^−6^ mol L^−1^ nanoparticles) with 1.0 mol L^−1^ NaCl to induce aggregation on the MNP@QD and to increase the size of the particles’ fluorescence cone. That would allow us to detect the MNP@QD with the available instruments in our facilities.

Since NaCl treatment could change the particles’ effects on BMDCs, we decided to repeat the previous assays (cytotoxicity evaluation, cytokine induction), replacing the MNP@QD with the MNP@QD containing NaCl (aforementioned as MNP@QD + NaCl). Although this aggregated form of the MNP@QD showed increased toxicity on the cells at the 5 × 10^−9^ and 10^−8^ mol L^−1^ doses, the effect on cytokine induction was similar to the “native” MNP@QD, as shown in Supplementary Material, Figure S4. We then performed fluorescence microscopy with MNP@QD + NaCl at the concentration of 10^−8^ mol L^−1^.

**Figure 7:**
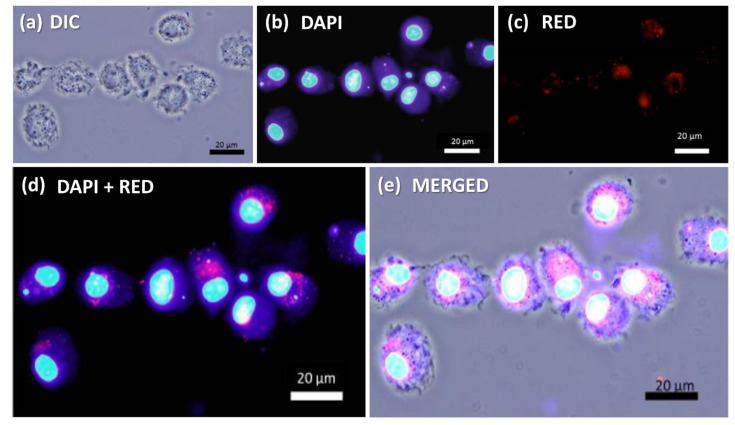
Fluorescence microscopy. (**a**) Differential Interference Contrast (DIC) image of BMDCs. (**b**) Blue-fluorescent DNA stain (DAPI; 4′,6-diamidino-2-phenylindole) that exhibits enhancement of fluorescence upon binding to AT regions of dsDNA. (**c**) Red channel under blue excitation of the MNP@QD. (**d**,**e**) Merged images.

Although this dose is more toxic, we wanted to increase the possibility of detecting the particles inside the cells. As shown in Figure 7, there is a faint nanoparticle signal at the DAPI channel and a remarkable signal at the red channel. Looking at the merged figure, it is possible to see that the particles acquire a perinuclear localization. To confirm the findings from Figure 7, we repeated the experiment, but we analyzed the cells in a confocal microscope this time. We included samples treated with the MNP@QD + NaCl at the 10^−9^ mol L^−1^ dose. As shown in Supplementary Material, Figure S5 and videos show confocal images from BMDCs treated with either 10^−9^ or 10^−8^ mol L^−1^. Taken together, these results clearly show that the nanoparticles are endocytosed during the period of 24 h incubation. The investigation of the mechanism of endocytosis is being carried out.

## 4. Discussion

Magnetic nanoparticles show great potential for cancer therapy and diagnosis. The great flexibility in the formulation strategies of the nanoparticles allows for the development of more specific therapies with fewer side effects [35]. In the present study, we tried to understand the potential and limitations of citrate-functionalized maghemite nanoparticles that were exploited as the anchoring surface to the fluorescent water-based cadmium telluride quantum dot by using cetyltrimethylammonium bromide. These multifunctional particles have the potential to work as both diagnostic tools (in MRI or fluorescence-based assays due to the QD properties) and as drug carriers. Synthesized initially as a tool for inspecting metal integrity for industrial purposes, our MNP@QD has excellent potential for biological applications [18]. The synthesis of MNP was based on the coprecipitation method following assisted oxidation. Controlling the reagent addition is essential to defining the nanoparticle structure. In conventional coprecipitation, when the Fe(III) and Fe(II) solutions are mixed, the significant difference in pH generates a contact interface.

If the local pH remains relatively low, the hydroxide ions will preferentially react with Fe(III) ions. Under such conditions, the initial favored phase will be akaganeite (β-FeOOH) [36]. However, rapid addition of base, or a rapid addition of iron salts to the base, will favor the formation of magnetite by generating Fe(OH)_2_ and FeOOH species. Even though this is an inverse spinel iron oxide with high loading magnetic content (Fe_3_O_4_), it is not a good candidate as a magneto-fluorescent building block since Fe(II) → Fe(III) intervalence transitions superimposed to the O^2−^ → Fe(III) charge-transfer band promotes its typical black color. This feature makes it unsuitable for magneto-fluorescent applications because the optical absorption and quenching by the FRET mechanism become rather critical, decreasing the luminescence intensity of the composite. For the reasons mentioned above, we have assisted the oxidation of magnetite to maghemite that is more stable in air and exhibits a much lower optical absorption in the visible region by using Raman spectroscopy, power diffraction, and infrared spectroscopy, as previously reported [18,19].

The use of cadmium quantum dots for biological applications has been the reason for great controversy. Cadmium is one of the most toxic heavy metals for the human organism, with the potential to harm the kidneys, the liver, and the heart [37,38]. It is understood that Cd quantum dots have great potential for generating ROS, including singlet oxygen, which exerts significant toxicity to the cells [39]. It was observed that MPA-coated QDs interacted with proteins in the cell culture medium and were internalized by clathrin-mediated endocytosis. The toxicity of CdTe to HEK293 cells was not due to the release of Cd ions after cell internalization but a consequence of ROS generation due to the nanoparticles’ big surface area and reactivity [40]. Our results show that the MNP@QD has little toxicity on the tumor cells, while the QD alone was very toxic (Figure 2a). The same pattern was observed when we evaluated the colony-forming capacity of the tumor cells treated with the MNP@QD, as shown in Supplementary Material, Figure S1. Thus, incorporating the QD at the surface of magnetic nanoparticles proved to be effective in reducing the toxicity of these heavy metal nanoparticles mostly because the quantum dots are trapped at the surface and are not allowed to be free in the cytoplasm promoting the ROS generation.

Evaluating the direct effects of the MNP@QD on the tumor cells was not enough, though, since the tumor microenvironment is composed of several types of cells that can directly shape the development of a tumor, like cancer-associated fibroblasts (CAFs), endothelial cells, pericytes, and immune cells like tumor-associated macrophages (TAMs), neutrophils, and others. Although the immune cells have the potential to destroy the tumor, they are often polarized towards a regulatory phenotype that favors tumor progression [41].

In a recent paper, Korangath et al., 2020, showed that starch-coated iron nanoparticles of ~100 nm are significantly enriched in tumors if conjugated with an antibody. Interestingly, the retention increased even if the antibody was not specific to any tumor antigen. Accordingly, the primary cells that captured the nanoparticles were immune cells like monocytes, macrophages, and dendritic cells [42]. Zanganeh et al., 2016, have shown that the off-label use of ferumoxytol, an FDA-approved nanoparticle used to correct iron deficiency, could inhibit the growth of adenocarcinomas in mice [43]. Co-cultures of macrophages with tumor cells in the presence of ferumoxytol showed that the immune cells were more cytotoxic and increased the expression of M1-related genes; M1 polarization was also observed in vivo [43]. We decided to study the MNP@QD effects on the activation status of dendritic cells. Although these cells are less frequent in the tumor microenvironment than the TAMs, they are crucial for priming adaptative immune responses against tumors [32]. Zhu et al., 2014, showed that TiO_2_ and Fe_3_O_4_@TiO_2_ could slightly induce the maturation of DCs and the secretion of TNF-α. Besides, both nanoparticles seemed to contribute to the internalization of the OVA antigen by BMDCs [44].

Other groups also showed the adjuvant capacity of iron nanoparticles in cross-priming. Luo et. al., 2019, showed that OVA complexes with PEGylated iron nanoparticles could increase the frequencies of DCs expressing CD40 and CD86, and induce more TNF-α [45]. Vaccines made of the OVA-NP complexes showed protection in prophylactic and therapeutic protocols against the B16 melanoma expressing OVA (either growing subcutaneously or in the metastatic model). The capacity of magnetic nanoparticles to promote cross-priming also seems to be influenced by the coating’s physical properties since positively charged, 3-aminopropyltrimethoxysilane (APTS)-coated nanoparticles showed an enhanced capacity to induce OVA-specific CD8^+^ T cell activation compared to negatively charged nanoparticles [46].

Regarding quantum dots, a Cd/Se QD with a zinc sulfide shell and a carboxylic acid coating inhibits the LPS-induced expression of CD80/CD86 by dendritic cells. On the other hand, PEGylated CuInS2/ZnS QD can be internalized by DCs and induce the production of TNF-α while inhibiting the production of IL-6. This QD was well tolerated in vivo [47]. Our MNP@QD could not induce the secretion of pro-inflammatory cytokines IL-6, TNF-α, and IL-12p70 when administered alone, Figure 4. In combination with LPS, however, it increased the secretion of IL-6 and TNF-α at some doses compared to LPS alone.

On the other hand, the MNP@QD seemed to have a more significant impact on the secretion of IL-1β in different conditions. It stimulated the secretion of small amounts of the cytokine if LPS-primed BMDCs were treated with 10^−8^ mol L^−1^ of the MNP@QD for 30 min, Figure 5a. Additionally, a 24-h treatment of BMDCs with the nanoparticles increased the secretion of IL-1β when the cells were treated with LPS + Nigericin, Figure 5c. These results suggest that our MNP@QD can activate the inflammasome, which agrees with other published data.

The inflammasomes are cytosolic multimeric complexes formed mainly in myeloid cells activated by PAMPs or DAMPs. Most of these complexes are constituted by a Nod-like receptor (NLR), the apoptosis-associated speck-like protein containing a C-terminal caspase recruitment domain (ASC) and caspase-1 [2,33]. Our experiments focused on the study of the NLRP3 inflammasome, whose activation comprises two steps: the priming and the assembly phases. In the priming phase, a TLR ligand or TNF-α induces an NF-κB-dependent transcription of *Il1b* and *Nlrp3* genes. Then, the assembly phase can be triggered by several stimuli (i.e., ATP, urate crystals) with the consequential oligomerization of NLR, ASC, and caspase-1 [6,7]. Caspase-1 can promote the functional maturation of cytokines like IL-1β and IL-18 and induce pyroptosis by cleavage of gasdemin D (GSDMD) [48].

**Scheme 1:**
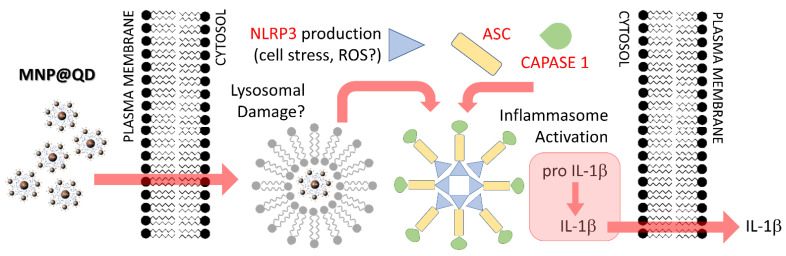
Illustrative representation of NLRP3 inflammasome activation in Murine Bone Marrow-derived Dendritic Cells after stimulation with MNP@QD, resulting in IL-1β secretion. Further studies intend to reveal the molecular mechanism of NLRP3 inflammasome activation by MNP@QD.

It has been known for over a decade that little particles can activate the NLRP3 inflammasome. Hornung et al., 2008, showed that the NLRP3 inflammasome is activated by cathepsin B after lysosomal rupture induced by either silica crystals or aluminum salts [34]. In line with that, Chen et al., 2018, found that PEI-coated iron nanoparticles could activate the inflammasome by a mechanism dependent on both lysosome rupture and ROS production. Interestingly, the authors tested iron nanoparticles of several sizes (30 nm, 80 nm, and 120 nm) and found that the smaller ones were better activators of the inflammasome [49]. In addition to the size, the shape and electric charges of nanoparticles influence the efficiency of inflammasome activation. Nanoparticles with sharp edges [50] are more prone to promote the rupture of the lysosomal membrane, and nanoparticles with negatively charged coatings [51] are better inflammasome activators. Our MNP@QD has a negative Zeta Potential, which is in line with the abovementioned data. Scheme 1 represents a general mechanism of NLRP3 inflammasome activation in murine bone marrow-derived Dendritic Cells after stimulation with MNP@QD, resulting in IL-1β secretion. Further investigation intends to elucidate the mechanism of action and the relative dependency of lysosomal rupture or ROS production in our model for the NLRP3 inflammasome activation.

Although great emphasis has been put on IL-1β’s possible effects on the tumor microenvironment and in the induction of antitumor immune responses, opportunities for treating cancer by inflammasome manipulation may be emerging due to the recent discovery of the potential of pyroptosis in inducing immune-mediated rejection of tumors. Wang et al., 2020, have developed a gold nanoparticle in which the N-terminal portion of gasdermin A3 (the cell membrane-penetrating fraction) is anchored to the particle by a compound that contains a *tert*-butyldimethyl silyl (TBS) group in its structure. This TBS group can be broken through a desylation reaction catalyzed by Phe-BF_3_, a compound typically used as a probe in positron emission tomography (PET), releasing the gasdermin fraction and triggering pyroptosis. The nanoparticles accumulated in the tumor site and the co-treatment of tumor-bearing mice with Phe-BF_3_ plus the nanoparticles induced pyroptosis in vivo. Apparently, only as few as 15% of the cells in the tumor underwent pyroptosis, but it was sufficient to promote tumor rejection. This tumor rejection was immune-mediated, as athymic nude mice and mice depleted of T CD4+, or T CD8+ cells lacked protection. Confirming the potential of inducing pyroptosis to treat tumors, Nadeem et al., 2021, synthesized an organosilica-coated iron oxide nanoparticle core and spiky manganese dioxide protrusions that efficiently caused pyroptosis in vitro and protected mice from a challenge with the breast tumor line 4T1 [52]. These results indicate the need for a closer look at how nanoparticles influence inflammasome activation. MNP@QDs clearly regulated the IL-1β production, indicating inflammasome activation. Whether these particles induce pyroptosis is worth investigating. Besides, due to nanoparticles’ great flexibility, we can modify our particles in the future to favor the activation of pyroptosis.

Finally, our MNP@QD proved useful for cell imaging since we could detect it inside the BMDCs. Since we only had access to conventional fluorescence and confocal microscopy, we could not detect the nanoparticles in their native state inside the cells. To overcome this problem, we treated the MNP@QD with 1.0 mol L^−1^ NaCl to induce aggregation among the particles. The emitted fluorescence is a function of the particle size, so the aggregated MNP@QD emitted more fluorescence than the individual particles. We could detect the aggregated nanoparticles in DCs treated with 10^−8^ mol L^−1^ MNP@QD with fluorescence microscopy and in DCs treated with 10^−9^ mol L^−1^ with confocal microscopy. Although the aggregation could produce artifacts and compromise the reliability of our data, we performed the same tests with the aggregated MNP@QD that were done with the native one. The aggregated MNP@QD behaved almost the same as the native particles regarding cytotoxicity and the induction of cytokine secretion, as shown in Supplementary Material, Figure S5, so we conclude that the aggregated nanoparticles probably interacted with the immune cells in the same way as the native ones. The current investigation intends to elucidate the mechanism of action and the relative dependency of lysosomal rupture or ROS production in our model for the NLRP3 inflammasome activation. It intends to unravel the mechanism by which MNP@QD activates the inflammasome, as well as the mechanism of internalization of MNP, QD, and MNP@QD [53,54,55,56].

## 5. Conclusions

Herein, we showed that citrate-functionalized maghemite nanoparticles that were exploited as the anchoring surface to the fluorescent water-based cadmium telluride quantum dot by using cetyltrimethylammonium bromide, MNP@QD, of ~45 nm in diameter, trigger NLRP3 inflammasome activation and subsequent release of proinflammatory interleukin IL-1β by murine bone marrow-derived dendritic cells, mainly if the myeloid cells have the chance to endocytose the particles prior to receiving inflammatory stimulation. Furthermore, our composite proved to be useful for cell imaging. Further studies intend to reveal the molecular mechanism of NLRP3 inflammasome activation by MNP@QD.

## Data Availability

All the raw data of this research can be obtained from the corresponding authors.

## Supplementary Materials

The following supporting information can be downloaded at: https://www.mdpi.com/article/10.3390/nano12183145/s1, Figure S1: (a) Representative RGB spectrum in which the red, green and blue components are monitored as a function of the reaction time for our synthetic parameters. (b) Correlation between the variation of the green and red component, the QD mean size, and their luminescence properties. This correlation plot was explained in detail in a previous paper [18]. Figure S2: (a) Statistical size distribution of QD. Inset: QD colloid photo under 470 nm excitation (b) Statistical size distribution of MNP. Inset: MNP colloid photo under visible light excitation. (c) Statistical size distribution of MNP@QD. (d) Time lapse of MNP@QD under 1.1 T external magnetic field and 470 nm excitation. The blue background is originated by the acetone solvent. Figure S3: (a) Toxicity evaluation of MNP, QD and MNP@QD in colony forming assays. Figure S4: (a) Toxicity evaluation of MNP@QD + NaCl. (b–h) Triggering study of IL-6, TNF-a and IL-12p70 cytokines secretion before and after LPS 24 h incubation. (i) Triggering study of IL-1b using LPS, MNP@QD (24 h of incubation) and Nigericin. Figure S5: Confocal microscopy. (a,e,i) Differential Interference Contrast (DIC) image of BMDCs in different concentrations. (b,f,j) Blue-fluorescent DNA stain (DAPI; 4′,6-diamidino-2-phenylindole) that exhibits enhancement of fluorescence upon binding to AT regions of dsDNA in different concentrations. (c,g,k) Red channel under blue excitation of the MNP@QD in different concentrations. (d,h,l) Merged images.
