# Supplementary material for: Quantum-Dot-Based Iron Oxide Nanoparticles Activate the NLRP3 Inflammasome in Murine Bone Marrow-Derived Dendritic Cells
Source: Nanomaterials (Basel). 2022 Sep 10;12(18):3145. doi: 10.3390/nano12183145 (PMC9502261; doi:10.3390/nano12183145)
Supplement: Supplementary file 1 [file nanomaterials-12-03145-s001.zip › nanomaterials-1892406-supplementary.pdf]

Supplementary Material

Quantum-Dot-Based Iron Oxide Nanoparticles Activate the NLRP3 Inflammasome in Murine Bone Marrow-Derived Dendritic Cells

Fernando Menegatti de Melo [1,2,†,*], Karine Kawasaki [3,†], Tarciso Almeida Sellani [3], Bruno Souza Bonifácio [3], Renato Arruda Mortara [3], Henrique Eisi Toma [1], Filipe Menegatti de Melo [3,*] and Elaine Guadelupe Rodrigues [3]

1 Department of Chemistry, Institute of Chemistry, University of São Paulo (USP), Av. Prof. Lineu Prestes 748, Butantã, São Paulo 05508-000, SP, Brazil
2 Metal-Chek do Brasil Indústria e Comércio, Research & Development Department, Rua das Indústrias, 135, Bragança Paulista 12926-674, SP, Brazil
3 Department of Microbiology, Immunology and Parasitology, Federal University of São Paulo (UNIFESP), Rua Botucatu, 862, Vila Clementino, São Paulo 04023-062, SP, Brazil
* Correspondence: fernando.menegatti.melo@usp.br (F.M.M.); fizao37@gmail.com (F.M.d.M.)
† These authors contributed equally to this work.

Real-time kinetics of QD formation

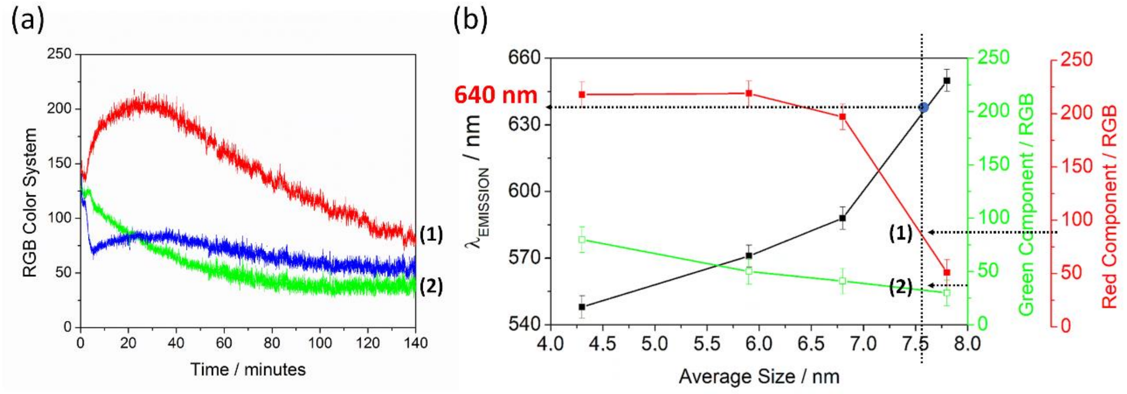

Figure S1. (a) Representative RGB spectrum in which the red, green and blue components are monitored as a function of the reaction time for our synthetic parameters. It was highlighted the final red component value (1), 85, and the final green component value, (2), 40. (b) Correlation between the variation of the green and red component, the QD mean size, and their luminescence properties. As we can see, the values (1) and (2) corroborates to 640 nm emission (very close to the experimental value, 649 nm) – this correlation plot was explained in detail in a previous paper [18].

Statistical size distribution of MNP, QD and MNP@QD

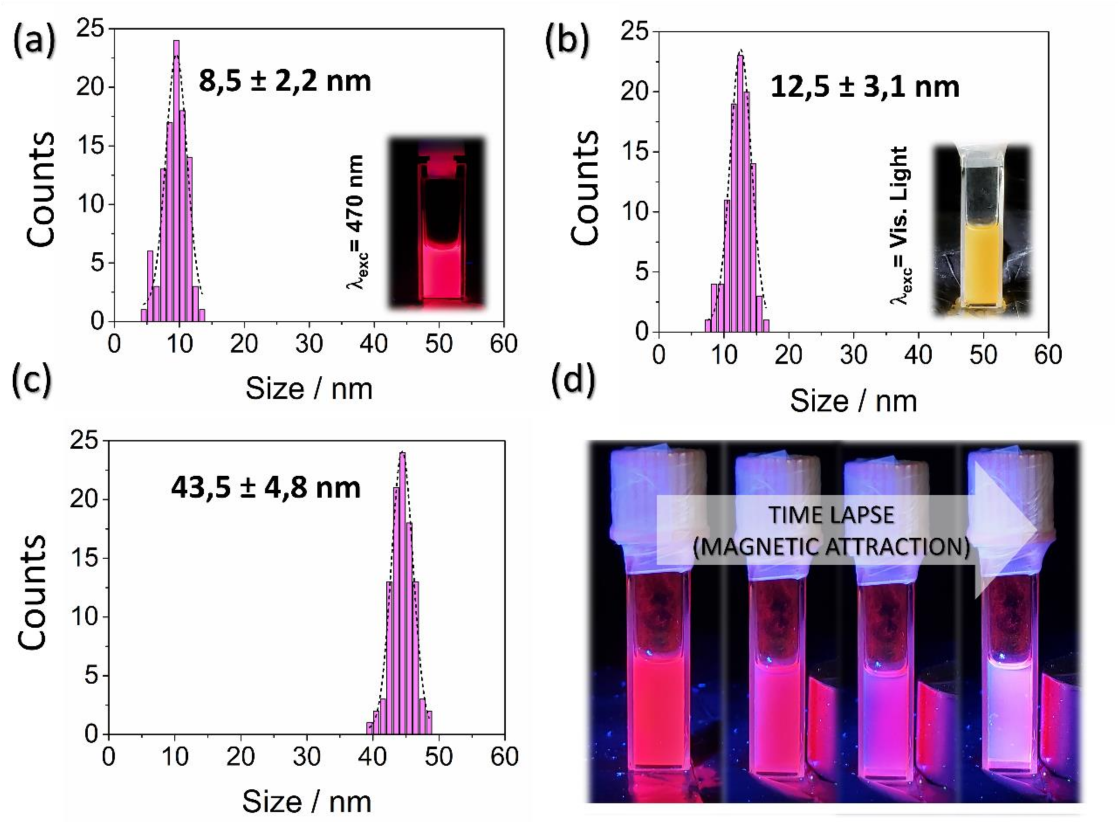

Figure S2. (a) Statistical size distribution of QD. Inset: QD colloid photo under 470 nm excitation (b) Statistical size distribution of MNP. Inset: MNP colloid photo under visible light excitation. (c) Statistical size distribution of MNP@QD. (d) Time lapse of MNP@QD under 1.1 T external magnetic field and 470 nm excitation. The blue background is originated by the acetone solvent.

Toxicity evaluation of MNP, QD and MNP@QD in colony-forming assay

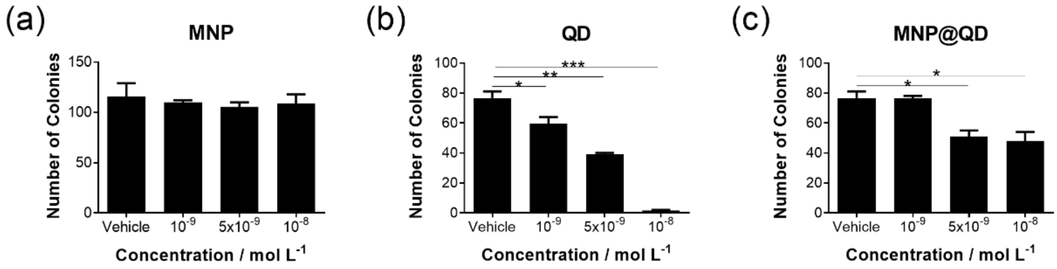

Figure S3. Toxicity evaluation of MNP (a), QD (b) and MNP@QD (c) in colony-forming assays. Each bar represents the average of four replicates. Error bars represent the standard error of the mean (SEM). Graphs are representative of two independent experiments.

Characterization of MNP@QD+NaCl in BMDCs

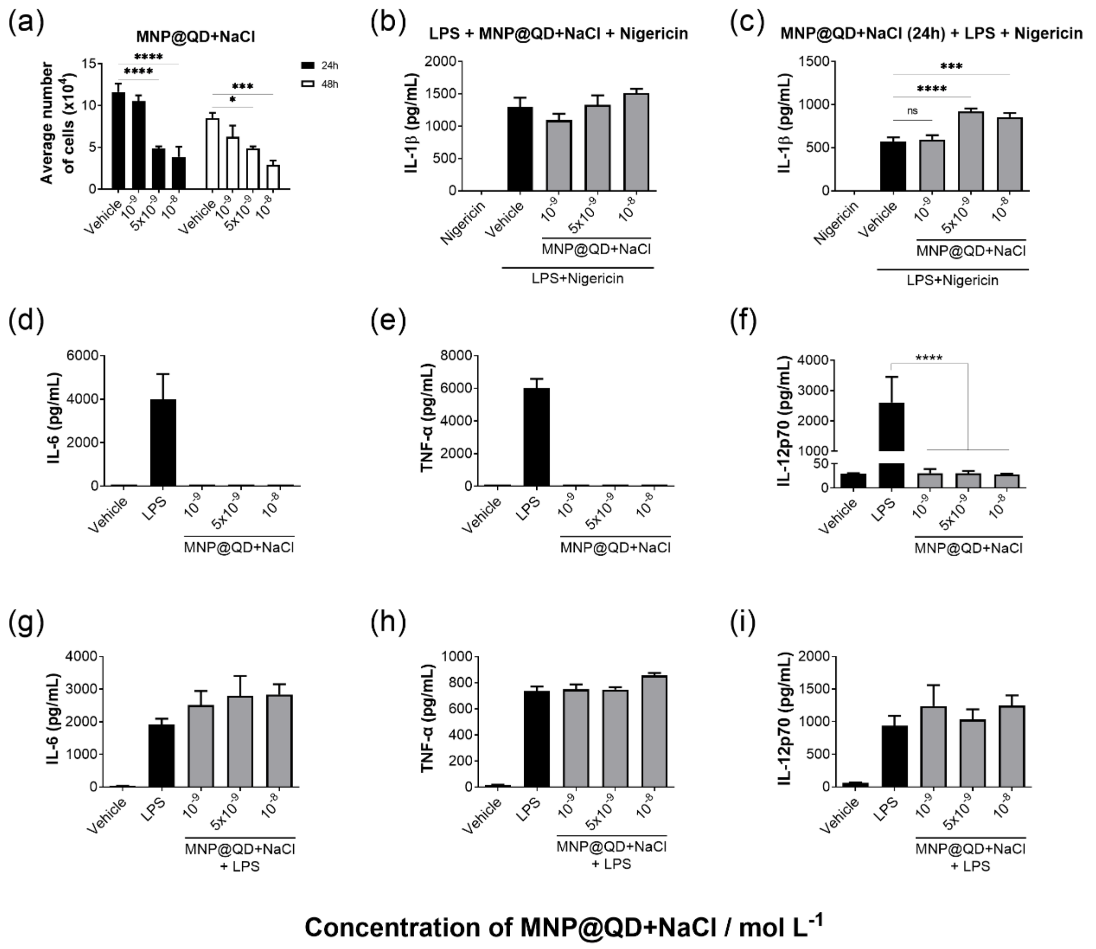

Figure S4. (a) Toxicity evaluation of MNP@QD+NaCl. (b-c) MNP@QD+NaCl effect on IL-1β secretion when added after (b, for 30min) or before (c, for 24h) LPS priming. (d-f) Evaluation of MNP@QD+NaCl induction of IL-6 (d), TNF-α (e) and IL-12p70 (f) when administered alone to BMDCs for 24h. (g-i) Induction of the same cytokines in BMDCs treated with MNP@QD+NaCl for 24h and then with LPS for 24h. Each bar represents the average of four replicates. Error bars represent the standard error of the mean (SEM). Graphs are representative of two independent experiments.

Confocal images of MNP@QD + NaCl in BMDCs

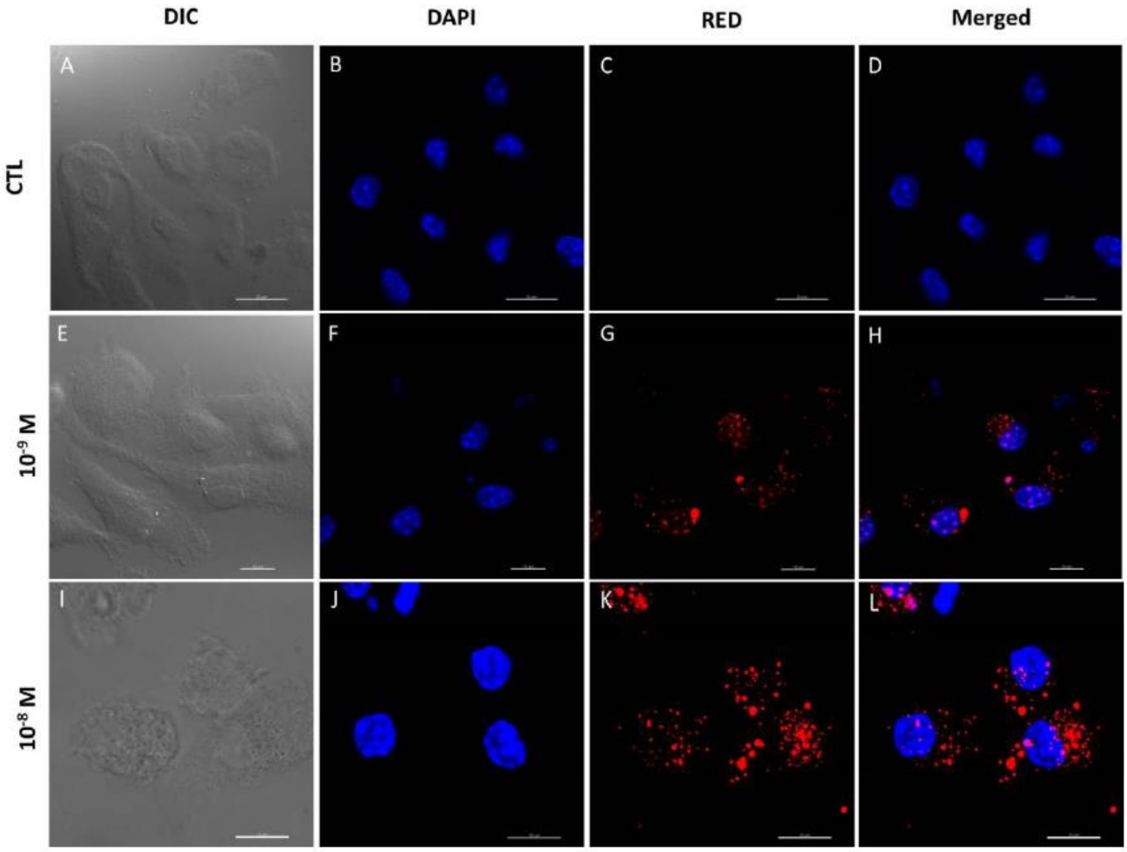

Figure S5. Confocal microscopy. (a, e, i) Differential Interference Contrast (DIC) image of BMDCs in different concentrations. (b, f, j) Blue-fluorescent DNA stain (DAPI; 4′,6-diamidino-2-phenylindole) that exhibits enhancement of fluorescence upon binding to AT regions of dsDNA in different concentrations. (c, g, k) Red channel under blue excitation of the MNP@QD in different concentrations. (d, h, l) Merged images. Scale bar: 20 μm
